# Alopecia areata: from immunopathogenesis to emerging therapeutic approaches

**DOI:** 10.3389/fimmu.2025.1681163

**Published:** 2025-11-07

**Authors:** Su-Young Kim, Hyun Joo Lee, Jihye Heo, Beom Joon Kim, Joon Seok

**Affiliations:** 1 Department of Dermatology, Chung-Ang University Hospital, Chung-Ang University College of Medicine, Seoul, Republic of Korea; 2 Department of Medicine, Graduate School, Chung-Ang University, Seoul, Republic of Korea; 3 Biomedical Research Institute, Chung-Ang University Hospital, Seoul, Republic of Korea

**Keywords:** alopecia areata, immunopathogenesis, emerging therapy strategies, immune cells, therapeutic targets

## Abstract

Alopecia areata (AA) is a non-scarring inflammatory hair loss disorder characterized by a T-cell–mediated autoimmune disease that targets the hair follicles. In particular, Natural Killer Group 2 member D (NKG2D)^+^CD8^+^ T cells have been identified as central players in its pathogenesis. Current treatment options have limited efficacy and are often associated with adverse effects and high risk of relapse upon discontinuation, highlighting the need for targeted and durable therapeutic strategies. Janus kinase (JAK) inhibitors have emerged as representative therapies; however, they are limited by a high relapse rate after treatment cessation. Recently, novel therapeutic approaches such as neutralizing antibodies targeting cytokines and chemokines, and sphingosine-1-phosphate (S1P) receptor modulators have gained attention. Various molecular markers associated with AA have been identified as potential therapeutic targets. This review provides a comprehensive overview of the roles of immune cells in AA pathogenesis and introduces emerging immunomodulatory strategies and novel therapeutic targets.

## 1 Introduction

Alopecia areata (AA) is an autoimmune disorder that induces nonscarring hair loss on hair-bearing surfaces, most commonly on the scalp. Its onset is often sudden and unpredictable. It is a relatively common condition, affecting up to 2% of the global population and can occur at any age (1). Clinical presentations range from solitary well-demarcated patches to more extensive patterns. Based on the extent of hair loss, AA is classified into subtypes such as alopecia totalis (AT, loss of all scalp hair) and alopecia universalis (AU, loss of all body hair) (2). Genetic predisposition and environmental factors contribute to the development of this disease (3).

The proximal region (bulge) of a healthy hair follicle maintains an immune-privileged status by suppressing MHC class I expression, limiting the activity of Langerhans cells (LCs), and through the action of immunosuppressive cytokines and hormones. This immune privilege effectively prevents the infiltration of immune cells, such as CD8^+^ and CD4^+^ T cells as well as natural killer (NK) cells, thereby protecting the follicle from immune-mediated attacks (2, 4). In AA, the collapse of this follicular immune privilege is considered a key pathogenic mechanism. Proinflammatory cytokines, including IFN-γ, TNF, IL-12, IL-15, and IL-18, along with cytotoxic molecules like granzyme B (GzmB) and perforin, play crucial roles in the progression of AA (5–7). These immune mediators disrupt follicular immune privilege and induce premature transition of the hair cycle into the catagen or telogen phase. Consequently, they promote immune cell–mediated attacks on the hair bulb, leading to hair loss and AA lesion formation (2).

Treatment options for AA include diphenylcyclopropenone (8), glucocorticoids (9), methotrexate (10), and cyclosporine (11). Excimer laser therapy has also been used as an adjunctive treatment (12). In recent years, small-molecule drugs, such as Janus kinase (JAK) inhibitors have been introduced as therapeutic agents (13). However, these treatments are often associated with disease relapse, limited efficacy, and low patient adherence, highlighting the need for more effective and sustainable therapeutic options. In this review, we discuss the immune mechanisms underlying AA and examine newly proposed therapeutic strategies and potential targets based on recent findings.

## 2 Pathogenesis of AA: hair follicle immune privilege and its collapse

The proximal region of hair follicles in healthy individuals, particularly the bulge area, is considered an immune-privileged site characterized by the near absence of CD4^+^ and CD8^+^ T cells. This immune privilege is maintained through multiple mechanisms, including downregulation of MHC class I expression, functional impairment of LCs, and the local production of immunosuppressive molecules such as pro-opiomelanocortin (POMC), transforming growth factor (TGF)-β1, TGF-β2, and macrophage migration inhibitory factor (MIF). In addition, immunoinhibitory signals, including vasoactive intestinal peptide receptor (VIPR) and CD200, further contribute to the maintenance of this immune-tolerant microenvironment. These mechanisms play a critical role in protecting hair follicles from the immune system and maintaining normal hair growth and cycling (2, 4).

**Figure 1:**
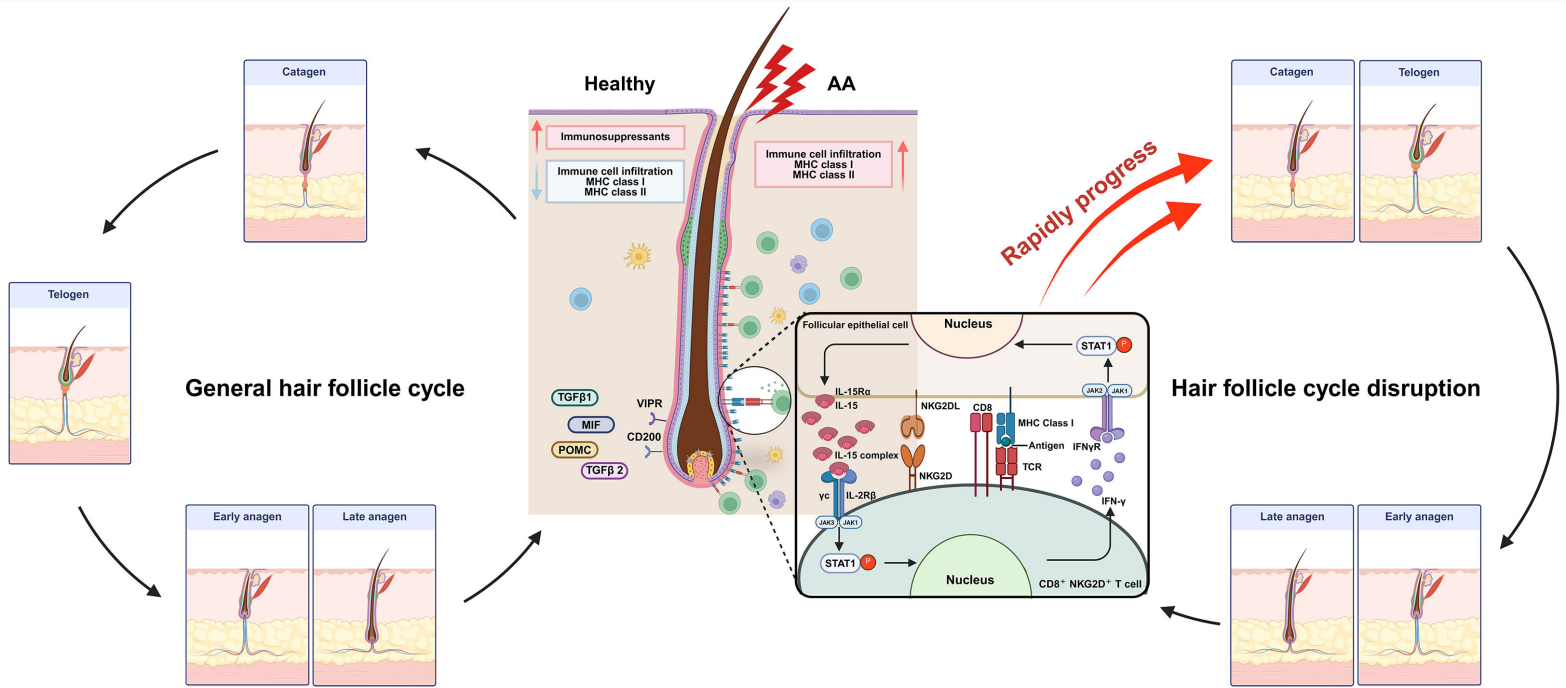
In healthy hair follicles, immune privilege is maintained through the low expression of MHC class I and class II molecules, along with high expression of immunosuppressive factors such as MIF, POMC, TGF-β1, and TGF-β2. Additionally, immunoinhibitory signals, including CD200 and the VIPR, suppress immune cell infiltration. However, in alopecia areata (AA), aberrant upregulation of MHC class I and II molecules leads to antigen recognition and increased infiltration of immune cells. Notably, IL-15 stimulation of pathogenic NKG2D^+^CD8^+^ T cells promotes direct follicular destruction via NKG2D/NKG2DL interactions. Furthermore, IFN-γ produced by these effector T cells activates follicular epithelial cells, triggering a pathogenic positive feedback loop that exacerbates disease progression. Late anagen hair follicles in patients with AA prematurely transition into the catagen phase, followed by a sustained telogen phase, ultimately resulting in hair loss. MIF, Macrophage migration inhibitory factor; POMC, pro-opiomelanocortin; VIPR, Vasoactive intestinal peptide receptor. Created in BioRender (Agreement number: XR28LF491U).

The collapse of immune privilege is a key pathogenic mechanism in AA. Although the precise cause has not yet been identified, environmental stress (14), epigenetic factors (15), and genetic predisposition (16) are thought to contribute to the disruption of immune privilege. Once this collapse occurs, inflammatory immune cells infiltrate the area and attack hair bulbs. During this process, the expression of MHC class I and II molecules increases in follicular and dendritic cells, leading to the infiltration of immune cells, such as CD8^+^ T cells, CD4^+^ T cells, and Mast cells (MC), into the hair follicle and its surrounding areas, thereby initiating an autoimmune response, which is elaborated upon in next section. Consequently, the anagen phase is shortened, a premature transition into the catagen phase occurs, and the telogen phase is prolonged, ultimately leading to the development of AA (2, 17) (**Figure 1**).

## 3 Immune cell–mediated mechanisms of AA

### 3.1 CD8^+^ T cells

CD8^+^ T cells are a key subset of cytotoxic T lymphocytes within the adaptive immune system and play a critical role in the defense against pathogens, such as viruses, bacteria, and tumors (18, 19). CD8^+^ T cells expressing Natural Killer Group 2 member D (NKG2D) play a pathogenic role in AA by directly identifying and attacking hair follicle cells. Xing et al. (20) showed that NKG2D^^+^^ CD8^^+^^ T cells are significantly increased in the skin and skin-draining lymph nodes (SDLNs) of AA mouse models, and transplantation of these cells into healthy mice induced AA-like lesions. Also, these CD8^+^ T cells produce interferon-gamma (IFN-γ) via the JAK1 and JAK2 signaling pathways, which in turn stimulates follicular dermal sheath cells to secrete IL-15 (20). IL-15 then further activates CD8^+^ T cells through the JAK1 and JAK3 pathways, promoting additional IFN-γ production, thereby establishing a positive feedback loop that amplifies the disease pathology (21). In particular, in AA, some CD8^+^ T cells have been reported to persist in the skin as tissue-resident memory T (T_RM_) cells characterized by the expression of CD69, CD49a, and CD103 (22, 23).

In AA, the relationship between CD8^+^ T cell pathogenicity and clonality has long been a central topic of investigation. De Jong et al. (24) showed that CD8^+^ T cell clonality was significantly increased at AA onset in a skin graft model using C3H/HeJ mice. In human patients with AA as well, clonally expanded T-cell clones have been detected in both the blood and scalp, and the extent of clonal expansion has been shown to correlate with disease severity. More recently, a study by Dai et al. (25) demonstrated that a single clonotypic CD8^+^ T cell expressing a defined T cell receptor (TCR) αβ sequence was sufficient to induce AA, providing direct evidence of a causal relationship between T-cell clonality and disease pathogenesis. Additionally, TCR sequencing of T-cell samples collected from the scalp and peripheral blood before and approximately 6 months after tofacitinib treatment revealed that although the frequency of most expanded T-cell clones decreased after treatment, a subset of clones persisted (24). These findings support the hypothesis that, in AA and other inflammatory skin disorders, disease recurrence may be driven by the reactivation of residual clonally expanded T cells or T_RM_ cells upon re-exposure to antigens. Indeed, the presence of autoantibodies and T cells reactive to candidate autoantigens has been reported in patients with AA (26–28). Although the autoantigens involved in AA have not yet been precisely identified, several candidate autoantigens proposed to date have been derived from keratinocytes and melanocytes of the hair follicle (**Table 1**) (29). Elucidating the role of antigen-specific CD8^+^ T cells in hair follicle destruction remains a critical area of ongoing research.

**Table 1:** Candidate autoantigens associated with AA.

Expected origins	Candidate autoantigens	Reported species	References
Melanocyte	Tyrosinase-related protein 2 and Tyrosinase	Mouse	(134)
	Tyrosine hydroxylase	Human	(135)
	Melanoma antigen recognized by T cells 1 and Glycoprotein-100	Human scalp grafted mouse	(136)
Lower hair cortex of hair follicle	Keratin 31	Mouse	(137)
Outer root sheath of hair follicle	Keratin 16	Human	(28)
Suprabasal layers and the inner layers of hair follicle	Fibroblast growth factor receptor 3	Human	(138)
Inner root sheath of hair follicle	Trichohyalin	Human	(28)
		Canine	(139)
	Keratin 71	Mouse	(137)

Recently, we reported that CD8^+^ T cells derived from virtual memory (T_VM_) cells, characterized as CD44^super-high (s-hi)^ CD49d^low^ (CD44^s-hi^) CD8^+^ T cells, play a central role in AA pathogenesis (22). These CD44^s-hi^ CD8^+^ T cells were selectively enriched in the skin and SDLNs of a mouse model of AA, and their adoptive transfer alone was sufficient to induce AA, suggesting that they are critical pathogenic effectors. CD44^s-hi^ CD8^+^ T cells originate from T_VM_ cells and are activated through bystander mechanisms in the presence of upregulated IL-12, IL-15, and IL-18 during AA. These cells express high levels of NKG2D and produce large amounts of proinflammatory cytokines, such as IFN-γ and TNF, as well as cytotoxic molecules, including perforin and GzmB, indicating their functional role as potent effector T cells in AA. This suggests that AA can be triggered by bystander activation in response to cytokine stimulation, even in the absence of a specific autoantigen. Interestingly, CD44^s-hi^ CD8^+^ T cells exhibited reduced TCR diversity compared to conventional T_VM_ cells, implying that a limited number of T_VM_ cells with diverse TCR repertoires may infiltrate the skin and subsequently undergo clonal expansion through antigen-dependent or -independent mechanisms. This observation suggests that although cytokine-driven activation appears to be an important mechanism, the involvement of antigen-specific clonal selection during the early phase of AA cannot be entirely ruled out. Further studies are required to elucidate the detailed mechanisms underlying this phenomenon.

### 3.2 CD4^+^ T cells

CD4^+^ T cells play a crucial role in the formation and maintenance of CD8^+^ T-cell populations by activating antigen-presenting cells to enhance the initial CD8^+^ T-cell response and supporting the survival and persistence of memory CD8^+^ T cells (30). These cells can differentiate into various subsets, each producing specific cytokines that modulate immune activity. McElwee et al. (31) reported that subcutaneous injection of CD4^+^ T cells isolated from mice with AA into C3H/HeJ mice induced systemic AA, whereas the injection of CD8^+^ T cells led to localized AA.

Among the CD4^+^ T-cell subsets, Th17 cells were found to be increased in the lesional skin as well as the circulating peripheral blood of patients with AA (32). Furthermore, the Th17-associated cytokine IL-17 was significantly increased in the lesional skin of AA patients compared with healthy controls, and serum IL-17 levels were also positively correlated with disease severity (33).

The dysfunction of regulatory immune cells, which suppress excessive immune responses and maintain immune homeostasis, is also believed to play a critical role in AA pathogenesis. Regulatory T cells (T_reg_, CD4^^+^^CD25^^+^^FoxP3^^+^^), which suppress autoimmune reactions and controls inflammation both directly and indirectly, is a representative regulatory cell type. However, numerous studies have reported abnormalities in the number and function of T_regs_ in patients with AA (32, 34, 35). From a genetic perspective, genome-wide association studies of AA have identified single nucleotide polymorphisms (SNPs) in genes involved in T_reg_ activation and proliferation, including *CTLA4*, *ICOS*, *IL2RA* (CD25), and *Eos* (also known as Ikaros family zinc finger 4; *IKZF4*). Additionally, SNPs in the promoter regions of *FOXP3* and *ICOSLG* have been associated with increased susceptibility to AA, suggesting that genetic defects in T_reg_ pathways may contribute to disease development (36, 37). However, reports on T_reg_ frequency in AA have been inconsistent. Some studies have found reduced proportions of T_regs_ in the skin of patients with AA or in AA mouse models, whereas others have reported no significant difference. Similarly, studies on peripheral blood mononuclear cells (PBMCs) have shown conflicting results, with some indicating normal T_reg_ frequency but impaired suppressive function, whereas others have observed increased T_reg_ proportions. Notably, some studies have reported an increased proportion of T_regs_ in SDLNs (23, 32, 38, 39). Functional abnormalities have also been observed. For example, reduced CD39 expression on T_regs_ in PBMCs from patients with AA has been linked to diminished immunosuppressive capacity. Additionally, decreased levels of TGF-β in patient serum have been reported (34, 35). However, owing to inconsistencies among studies, further well-designed and comprehensive studies are needed to clarify the precise role and functional status of T_regs_ in the pathogenesis of AA.

### 3.3 γδ T cells

It is well established that NKG2D^+^ cells play a pivotal role in the pathogenesis of AA. NKG2D is expressed not only on CD8^+^ T cells and NK cells but also on human γδ T cells (40). A recent study by Uchida et al. (41) has demonstrated that γδ T cells are sparsely present in healthy human scalp skin, with the majority belonging to the Vδ1^+^ subset. These cells exhibit a non-activated phenotype (CD69^-^NKG2D^dim^) and express receptors for CXCL10 and CXCL12. They are primarily localized within and around the hair follicle infundibulum. In marked contrast, a significant increase in Vδ1^+^ γδ T cells was observed in the hair follicles of AA lesions, where these cells infiltrated both inside and around the suprabulbar and bulbar epithelium. They displayed a proinflammatory phenotype, characterized by upregulated expression of NKG2D and IFN-γ and downregulated CD200R, suggesting a potential role in amplifying pathogenic T-cell responses (41).

Notably, infiltration of similarly proinflammatory Vδ1^+^ T cells was also observed around non-lesional AA hair follicles, indicating that these cells may be involved in the early stages of AA pathogenesis.

### 3.4 Dendritic cells

Plasmacytoid dendritic cells (pDCs) are key producers of type I interferons (IFN-α/β) in response to viral stimuli and can secrete large amounts of IFN-α (42). They express CD123, HLA-DR, and BDCA-2 as well as Toll-like receptors TLR7 and TLR9, of which the latter mediates type I IFN production (43, 44). Recent studies have confirmed the presence of numerous pDCs surrounding hair follicles in patients with AA, as revealed by BDCA-2 immunostaining (45). Moreover, the injection of CpG-activated pDCs into healthy mice was shown to induce AA, with the accumulation of pDCs in non-lesional skin preceding visible hair loss (46). In addition, cases of AA have been reported following administration of TNF inhibitors such as adalimumab and infliximab. Under normal conditions, TNF suppresses the development of pDCs and thereby regulates IFN-α production. Neutralization of TNF may result in sustained IFN-α secretion by pDCs, leading to the breakdown of the immune privilege of hair follicles (47–50). These findings provide clinical evidence that pDCs can act as pathogenic immune cells during AA immunopathogenesis. The secreted IFN-α is thought to impair the immune privilege of hair follicles and induce the expression of CXCL10/IP-10, thereby promoting the recruitment of CXCR3^+^ Th1 and CD8^+^ T cells to the hair bulb, contributing to the early immunopathogenesis of AA (46).

The infiltration of CD1a^+^ LCs into the skin of patients with AA may also play a critical role in disease pathogenesis. The number of CD1a^+^ LCs was significantly increased in the epidermis, upper follicular, and deep perifollicular areas of patients with AA, with even greater infiltration observed in the epidermis and deep perivascular and perifollicular regions of patients with active hair loss. A significant positive correlation was observed between LCs in the deep perivascular areas and CD8^+^ T cells in the upper perifollicular areas, and a strong correlation was observed between LC infiltration in the upper perivascular areas and CD8^+^ T cells in the deep perifollicular regions in active cases. These findings suggest that LCs contribute to the activation of CD8^+^ T cells by mediating a cytotoxic immune response against hair follicles (51).

Taken together, these results indicated that both pDCs and LCs act as key initiators of immune activation in AA lesions and may promote hair follicle destruction through the activation of CD8^+^ cytotoxic T lymphocytes.

### 3.5 Mast cells

Several studies have shown that MCs are markedly increased in both number and activity in the lesional skin of patients with AA, suggesting that MCs may play a more direct role in AA pathogenesis (51, 52). In particular, the accumulation and degranulation of perifollicular MCs, along with their altered expression profiles, suggest an active role in the immune dysregulation underlying AA (52).

In healthy human skin, MCs are predominantly non-degranulated and express stem cell factor receptor c-Kit and MHC class I molecules. They also maintain immunoinhibitory function by producing cytokines such as IL-10 and TGF-β1. In contrast, lesional AA skin exhibits a phenotypic shift in MCs, with reduced IL-10 and TGF-β1 levels and upregulation of co-stimulatory molecules including OX40L, CD30L, 4-1BBL, and ICAM-1. This switch from an immunosuppressive to a proinflammatory phenotype fosters an environment conducive to autoimmune activation, particularly by promoting pathogenic CD8^+^ T-cell responses (52).

Direct contact between MCs and CD8^+^ T cells is frequently observed in AA lesions, and a positive correlation has been reported between the number of MCs and CD8^+^ T-cell infiltration in the deep perifollicular region (51). During these interactions, most MCs express OX40L and, in some cases, 4-1BBL or ICAM-1, which may provide additional co-stimulatory signals that enhance CD8^+^ T-cell activation and proliferation. Notably, MCs in this context are actively degranulating, suggesting that CD8^+^ T cells may also be stimulated via the activation of PAR-2, a tryptase receptor. Together, these findings imply that the MC–CD8^+^ T-cell cross-talk serves as a critical axis in breaking hair follicle immune privilege and promoting autoimmune attack in AA. Such immunopathological features have been consistently observed in both human and murine AA models (52).

Importantly, clinical reports indicating that antihistamines may alleviate symptoms in some patients with AA support the hypothesis that MCs are functionally relevant contributors to the disease (53, 54). These findings highlight the potential of MCs as therapeutic targets in AA.

### 3.6 Innate lymphoid cells-type 1

Innate lymphoid cells-type 1 (ILC1) are a component of type 1 immunity that express NKG2D and recognize conserved phosphoantigens (55). They play a key role in antitumor immunity (56). The activating receptor NKG2D and its ligands (such as MICA and ULBP3) function as a critical receptor–ligand axis in the immune responses of NK cells, ILC1, γδ T cells, and CD8^+^ T cells, and are implicated not only in tumor immunity but also in various autoimmune diseases (57, 58).

Britva et al. (59) recently identified a significant increase in ILC1 cells surrounding both lesional and non-lesional hair follicles in patients with AA. Therefore, co-culture experiments were conducted using autologous ILC1-like cells (ILC1lc) and *ex vivo* organ-cultured, stressed healthy human scalp hair follicles. The results showed that ILC1lcs induced several hallmark features of AA; they significantly promoted premature, apoptosis-driven hair follicle regression (catagen), follicular cytotoxicity, dystrophy, and, most notably, the collapse of the physiological immune privilege of hair follicles. Moreover, when activated NKG2D^+^/IFN-γ^+^ ILC1lcs were intradermally injected into human scalp skin xenografted onto SCID/beige mice, they rapidly induced AA-specific lesions. These findings demonstrate that ILC1 cells, as innate immune effectors, can act as pathogenic drivers in AA, highlighting their potential role in disease initiation independent of adaptive immunity.

### 3.7 Eosinophils

Eosinophils are innate immune cells primarily involved in parasitic infections and allergic reactions. They can secrete a variety of inflammatory cytokines, enzymes (e.g., major basic protein [MBP], eosinophil cationic protein [ECP]), and reactive oxygen species, thereby contributing to tissue damage and amplifying immune responses. Eosinophils have been reported to be involved in the pathogenesis of autoimmune and chronic inflammatory diseases through these functions, and their role in AA has also been studied (60, 61).

Some studies have reported that eosinophilic infiltration around the hair bulb or within the fibrous tracts is a relatively common histopathological finding across all stages of AA and have suggested that it could be a useful diagnostic marker for distinguishing AA from other types of non-scarring alopecia (62, 63). However, other studies have shown that eosinophil infiltration is rarely observed in AA lesions (64). In particular, in cases of chronic AA where peribulbar lymphocytic infiltration is not prominent—making it difficult to differentiate it from androgenetic alopecia (AGA)—the frequency of eosinophilic infiltration has been reported to be as low as 7.1%, with no significant difference from that observed in AGA or trichotillomania (65).

These conflicting findings indicate that the presence and pathological significance of eosinophils in AA remain unclear. It is not yet known whether eosinophil infiltration represents a secondary phenomenon limited to certain patients or is a central component of disease pathogenesis. Further studies with larger cohorts and more refined analyses are required to elucidate the role of eosinophils in AA.

### 3.8 Invariant natural killer T cells

Invariant natural killer T (iNKT) cells are a unique subset of T lymphocytes that express semi-invariant T-cell receptors (TCRs) and recognize lipid antigens presented by CD1d (66). These cells have recently attracted attention owing to their immunoregulatory functions in autoimmune diseases. In a humanized mouse model of AA, activation of iNKT10 cells by α-galactosylceramide (α-GalCer) led to increased IL-10 production and promoted hair regrowth (67). These findings suggest that iNKT10 cells have both preventive and therapeutic potential in AA, and may serve as promising targets for future immunomodulatory therapies.

## 4 Emerging and experimental therapies

### 4.1 JAK inhibitors

JAK inhibitors, based on their immunosuppressive effects, have been widely used to treat various autoimmune and inflammatory diseases such as atopic dermatitis, psoriasis, and rheumatoid arthritis, and their efficacy and safety have drawn significant attention (68). Recently, the official approval of both oral and topical JAK inhibitors has expanded their range of applications. Therefore, their potential as treatments for AA has been actively explored.

Harel et al. (69) reported that pharmacological inhibition of the JAK–STAT signaling pathway can induce hair growth. In a C57BL/6 mouse model, the topical application of a JAK inhibitor to hair follicles in the telogen phase led to a rapid and uniform transition to the anagen phase within 7–10 days, promoting hair growth. Histological observations and gene expression analyses revealed activation of hair growth–related pathways, along with increased proliferation of hair follicle progenitors and stem cells. In addition, in a human scalp xenograft model and hair follicle organ culture system, JAK inhibitors significantly increased the rate and density of hair growth, and enhanced the hair-inducing ability of three-dimensionally cultured human dermal papilla cells. These findings demonstrate that JAK inhibitors not only exert immunosuppressive effects but also play a positive role in promoting hair growth, making them leading therapeutic candidates for severe AA.

Baricitinib, a JAK1/2 inhibitor, became the first oral drug approved by the Food and Drug Administration (FDA) in 2022 for the treatment of severe AA in adults. In two phase 3 clinical trials, BRAVE-AA1 (NCT03570749) and BRAVE-AA2 (NCT03899259), adult patients with a SALT score of 50 or higher were administered baricitinib, and the proportion of patients who achieved a SALT score of 20 or lower after 52 weeks was assessed (70). In BRAVE-AA1, the response rates were 40.9%, 21.2%, and 4.1% for the 4-mg 2-mg and placebo groups, respectively. In BRAVE-AA2, the corresponding rates were 36.8%, 24.4%, and 5.3%, respectively, confirming the significant therapeutic effect of baricitinib. Furthermore, among patients who met their treatment goals at 52 weeks and continued baricitinib through 104 weeks, 90.7% (4 mg) and 89.2% (2 mg) maintained their response, demonstrating long-term efficacy (71). Reported side effects included acne, urinary tract infections, and elevated creatine kinase levels, whereas serious adverse events such as severe infections, herpes zoster, major cardiovascular events, and malignancies were rare (70, 72). A phase 3 trial investigating the efficacy, safety, and pharmacokinetics of baricitinib in pediatric patients aged 6 to <18 years is currently at the patient-recruitment stage (BRAVA-AA-PEDS; NCT05723198).

Deuruxolitinib (CTP-543) is a selective JAK1/2 inhibitor approved by the FDA in 2024 as an oral treatment for adults with severe AA. In the phase 3 clinical trials THRIVE-AA1 and THRIVE-AA2, patients with a SALT score of 50 or higher were administered deuruxolitinib, and the proportion of patients achieving a SALT score of 20 or less after 24 weeks was assessed. In THRIVE-AA1 (NCT04518995) (73), the response rates were 41.5% (12 mg), 29.6% (8 mg), and 0.8% (placebo), whereas in THRIVE-AA2 (NCT04797650), the corresponding response rates were 38.3%, 33.0%, and 0.8%, as reported on ClinicalTrials.gov. The reported side effects included headache, acne, and nasopharyngitis, whereas serious adverse events were rare (73). Long-term safety data are expected in future studies.

Ritlecitinib is the first oral medication to selectively inhibit JAK3 and TEC and was approved by the FDA in 2023 for the treatment of severe AA in patients aged 12 years and older. Unlike JAK1 and JAK2, which are widely expressed in various cell types, JAK3 is predominantly expressed in hematopoietic and lymphoid cells, offering higher immune cell selectivity, thereby reducing the risk of systemic side effects (74). In particular, JAK3 inhibition is not associated with side effects, such as anemia and thrombocytopenia, that may occur with JAK2 inhibition, suggesting favorable clinical utility (75). Dai et al. (76) demonstrated that the selective inhibition of JAK3 was sufficient to effectively treat AA. In a C3H/HeJ AA mouse model, treatment with a JAK3 inhibitor led to 100% hair regrowth, reduced CD8^+^ T-cell infiltration, and suppressed MHC expression. Topical application of the JAK3 inhibitor also showed significant therapeutic effects. RNA-sequencing analysis of skin biopsies revealed downregulation of inflammation-related genes such as IFN-γ, granzyme B, and perforin, along with improvement in the Alopecia Areata Disease Activity Index score. Furthermore, in a mouse model treated with ifidancitinib, a JAK1/3 inhibitor, an increased proportion of CD44^+^ effector T cells expressing high levels of co-inhibitory receptors, such as PD-1 and TIM-3, was observed, indicating the induction of T-cell exhaustion (77). This finding is consistent with previous research by Mayack et al. (78), who reported increased PD-1 and LAG-3 expression in JAK3 knockout T cells with defective γc cytokine signaling. These results suggest that JAK3 inhibition contributes to AA treatment not only by suppressing T-cell proliferation, differentiation, and survival, but also by promoting T-cell exhaustion. However, because exhaustion may compromise the protective functions of immune cells, further investigation is required to assess its long-term implications. Clinically, in the ALLEGRO phase 2b/3 trial (NCT03732807) involving patients aged 12 years and older with a SALT score of 50 or greater, ritlecitinib treatment showed the highest response rate in the group that received a 200 mg induction dose followed by a 50 mg maintenance dose, with 31% of patients achieving a SALT score of 20 or lower at week 24. Most ritlecitinib-treated groups showed a statistically significant therapeutic effect compared with the placebo group (79). In contrast, the subsequent open‐label study ALLEGRO‐LT (NCT04006457), which included patients with a SALT score of 25 or greater, showed more favorable outcomes: continuous treatment with ritlecitinib over 24 months led to 73.5% and 66.4% of patients achieving SALT scores ≤20 and ≤10, respectively, based on observed values, and 60.9% and 53.5%, respectively, based on the last observation carried forward, confirming the sustained efficacy of the drug (80). Moreover, follow-up analyses of long-term response patterns revealed that among patients with no initial response, 11% eventually achieved SALT ≤20 after more than 1 year of continuous treatment. These findings underscore the importance of long-term therapy for AA and suggest that focusing solely on early treatment responses may underestimate the full potential of treatment (81).

The JAK inhibitors currently approved by the FDA for the treatment of AA include baricitinib, deuruxolitinib, and ritlecitinib, which are considered representative agents for this indication. Yan et al. (82) conducted a network meta-analysis (NMA) comparing the three JAK inhibitors—baricitinib, deuruxolitinib, and ritlecitinib. In this analysis, deuruxolitinib 12 mg/8 mg demonstrated superior therapeutic efficacy compared with other JAK inhibitors, particularly in patients with severe AA. However, it has also been reported to be associated with a higher incidence of adverse events than other highly selective JAK inhibitors. In contrast, the NMA data reported by Gupta et al. (83) indicated no significant difference in efficacy among treatment groups receiving deuruxolitinib 8 mg twice daily, ritlecitinib 50 mg once daily, and baricitinib 4 mg once daily. Although several indirect comparison studies have been conducted (82–84), direct head-to-head clinical trials comparing JAK inhibitors and other systemic agents currently used for the treatment of AA remain limited. Therefore, the results of NMAs should be interpreted with caution, taking into account methodological limitations such as differences in study design and heterogeneity in patient populations. Additional research is warranted to draw definitive conclusions regarding their relative efficacy and safety. In addition to approved therapies, several other JAK inhibitors are currently being investigated in clinical trials for AA treatment, although they have not yet received regulatory approval (85, 86).

Currently, a phase 3 clinical trial is underway evaluating upadacitinib (a JAK1 inhibitor) in adults and adolescents aged 12 years and older with severe AA (NCT06012240). According to a retrospective study reported by Li et al. (87), patients with a median SALT score of 73.9 ± 29.5 and a disease duration of 5 years received once-daily upadacitinib at doses ranging from 7.5 mg to 30 mg. At week 36, all the participants experienced either partial or complete hair regrowth. Similar to the effects observed upon baricitinib and ritlecitinib treatment, upadacitinib treatment demonstrated progressive improvement over time, and notable hair regrowth was observed even in some of the patients who had previously failed to respond to or relapsed after treatment with other JAK inhibitors (baricitinib, tofacitinib, and abrocitinib) for more than 3 months. These findings contribute to the high expectations of the outcomes of a phase 3 trial of upadacitinib. In addition, various JAK inhibitors are currently undergoing phase 2 or phase 3 clinical trials as potential treatments for AA. The key candidates include ivarmacitinib (SHR0302, JAK1 inhibitor, NCT05470413), jaktinib (JAK1/2/3 inhibitor, NCT05051761), brepocitinib (PF-06700841, JAK1/TYK2 inhibitor, NCT05076006), and deucravacitinib (BMS-986165, TYK2 inhibitor, NCT05556265). In addition, SYHX1901 (JAK/SYK inhibitor, NCT06562894) has not yet begun recruitment, whereas the status of KL130008 (JAK1/2 inhibitor, NCT05496426) is unknown, with no progress reported. Notably, ifidancitinib (JAK1/3 inhibitor) was the only candidate developed as a topical solution and had entered a phase 2 clinical trial (NCT03759340); however, the trial was terminated early owing to lack of efficacy. Similarly, tofacitinib (JAK1/2/3 inhibitor, NCT03800979) progressed to phase 4 clinical trials but ultimately did not receive FDA approval for the treatment of AA.

Although various JAK inhibitors are being developed, even among patients treated with baricitinib, which is FDA-approved for the treatment of AA, approximately 10% experience hair loss recurrence within 8 weeks of treatment discontinuation (88). By week 152, approximately 80% had lost treatment response, and among those who were retreated, only 63% in the 2-mg group and 87.5% in the 4-mg group regained their response. This suggests that continuous treatment with JAK inhibitors is necessary, even in patients who have achieved hair regrowth and highlights the need for additional therapeutic strategies to prevent AA relapse.

### 4.2 Low-dose IL-2

*In vivo*, IL-2 plays a critical role in maintaining the survival, proliferation, and functional stability of T_reg_ cells, highlighting its importance as a key regulator of T_reg_-mediated immune control. Notably, low-dose IL-2 has demonstrated therapeutic potential in several autoimmune diseases (89). T_reg_ cells express high-affinity IL-2 receptors, allowing them to effectively compete for limited IL-2 levels, and respond even to low concentrations. Therefore, precise dose control is essential when administering IL-2. Even slight increases in dose may lead to off-target immune activation, such as the stimulation of memory T cells or NK cells, which can result in unintended side effects. Additionally, IL-2 has a short half-life *in vivo* and requires frequent and repeated administration, which poses a practical limitation (90).

In a study by Castela et al. (91), subcutaneous injections of low-dose recombinant IL-2 in patients with severe AA refractory to previous systemic therapies led to T_reg_ cell recruitment into lesional scalp areas in 4 out of 5 patients, with 1 patient showing no response. In a subsequent multicenter randomized controlled trial conducted by Le Duff et al. (92), 43 adult patients with severe AA were followed for 52 weeks. Although IL-2 therapy significantly increased peripheral T_reg_ cell counts, no substantial hair regrowth was observed. The researchers suggested that this limited efficacy might be because of the expansion of naïve T_reg_ cells, which lack skin-homing capabilities. Similarly, in a murine model, administration of a cytokine complex composed of human IL-2, anti-IL-2 antibody, and mouse IL-2 Fc led to an 8–10-fold increase in T_reg_ cells, but failed to reverse established AA (93). These findings suggest that combination strategies, rather than IL-2 monotherapy, may yield better outcomes.

In summary, low-dose IL-2 therapy has the potential to treat AA by promoting T_reg_ cell expansion. However, its therapeutic effect as a monotherapy appears insufficient, highlighting the need for further studies to establish effective combination strategies and evaluate their clinical efficacy.

### 4.3 Immunomodulatory strategies targeting receptors, cytokines, chemokine

NKG2D is an activating receptor that mediates critical cytotoxic responses in AA pathogenesis. According to our preliminary studies, blockade of NKG2D using anti-NKG2D antibodies effectively prevented disease onset in an AA mouse model (22). Furthermore, *in vitro* experiments demonstrated that anti-NKG2D treatment significantly reduced the cytotoxic activity of IL-15–stimulated CD44^s-hi^ CD8^+^ T cells against YAC-1 target cells.

IL-2, IL-9, and IL-15 play pivotal roles in the activation of cytotoxic T and NK cells, thereby contributing significantly to AA immunopathogenesis. Antibody-based therapies targeting IL-2, IL-9, and IL-15 have previously been shown to alleviate AA symptoms in animal models (77). Recently, small-molecule inhibitors targeting these cytokines have been developed. EQ101 (formerly known as BNZ-1) is a novel therapeutic peptide that combines the high selectivity of monoclonal antibodies with the cytokine-inhibitory properties of small molecules. An open-label phase II clinical trial (NCT05589610) of EQ101 has been completed in adults with moderate-to-severe AA to evaluate its safety and efficacy (86, 94). These developments underscore the growing potential of novel therapeutic options targeting cytokine signaling in AA. Similarly, we also demonstrated that neutralizing antibodies targeting IL-12, IL-15, and IL-18, which are upregulated in AA, effectively prevented disease onset in mouse models (22). In addition, previous studies have reported that the antibody-based targeting of CXCL12 successfully prevents and treats AA (95), further supporting the potential of therapeutic strategies involving neutralizing antibodies in this disease.

### 4.4 Cell trafficking inhibition

Elevated levels of circulating CD8^+^ T cells and cytotoxic T cells are sustained during the chronic phase of AA (96), indicating that targeting T-cell activity and migration could be a promising therapeutic strategy for this disease.

Sphingosine-1-phosphate (S1P) and its receptor S1PR1 play a central role in directing immune cell migration (97, 98). S1P is maintained at relatively high concentrations in the blood and lymphatic fluid, whereas its levels are considerably lower in lymphoid tissues. The migration of lymphocytes is controlled by this S1P gradient that exists between the lymphoid organs and the circulatory system. In circulating naïve T-cells, S1PR1 is internalized upon exposure to high concentrations of S1P in the bloodstream, resulting in reduced surface expression of the receptor. Consequently, newly arriving naïve T cells in the lymph nodes are unable to detect the S1P gradient and remain sequestered within the lymph node. Following antigen stimulation, surface expression of S1PR1 is further suppressed for several days, preventing activated T cells from exiting the lymph node. Over time, S1PR1 is re-expressed—within minutes to hours in naïve T cells and several days in activated effector T cells—enabling them to once again sense the S1P gradient and migrate out of the lymph nodes (99). Among the five known S1P receptors (S1PR1–S1PR5), S1PR1 and S1PR4 are the most relevant in lymphocytes, where they regulate their exit from lymphoid organs. In recent years, substantial progress has been made in the development of selective S1P receptor modulators as immune-targeting therapies. Agents, such as fingolimod, siponimod, and ozanimod, have already been approved for treating multiple sclerosis and are being actively investigated in clinical studies for the treatment of other autoimmune conditions, including inflammatory bowel disease (97, 98).

Etrasimod, an oral modulator of S1PR1, 4, and 5, has recently shown clinical promise for the treatment of AA (100). Although 2-mg and 3-mg doses did not show a statistically significant effect in the double-blind phase, both demonstrated favorable trends in SALT score reduction compared with the placebo. In the open-label extension, continued administration over 52 weeks resulted in sustained clinical improvement, particularly in the 3-mg group. Notably, peripheral lymphocyte levels returned to near-baseline values within 4 weeks of discontinuation, suggesting that prolonged immunosuppression was unlikely. Meanwhile, the selective S1PR1 and 4 modulator NXC736 is currently undergoing clinical evaluation and recruiting participants for the treatment of AA (NCT06104839), and its selective targeting of only S1PR1 and S1PR4—receptors most closely associated with lymphocyte trafficking—highlights its potential as a more specific targeted therapy.

### 4.5 Dupilumab

Dupilumab is a fully human monoclonal antibody that binds to the interleukin-4 receptor alpha (IL-4Rα), thereby blocking IL-4 and IL-13 signaling pathways. It plays a crucial role in suppressing type 2 helper T cell (Th2)-mediated immune responses. This drug was approved by the U.S. FDA in 2017 for the treatment of moderate-to-severe atopic dermatitis (AD) in adult patients who do not respond adequately to topical therapies (101). Since then, its therapeutic potential has been explored for a range of type 2 inflammatory diseases.

AA is traditionally considered an autoimmune disorder primarily driven by Th1 responses and CD8^+^ T cells. However, recent findings suggest that Th2 immune activation may also occur in a subset of patients, prompting growing interest in Th2-targeted therapeutic strategies (102–105). Based on this hypothesis, dupilumab has been investigated for its potential efficacy in the treatment of AA, and an increasing number of clinical reports and case studies support this possibility. Penzi et al. (101) first reported a case in which a pediatric patient diagnosed with both AD and AT received dupilumab for 9 months, resulting in significant improvement of dermatitis and hair regrowth over more than 60% of the scalp. A large population-based study by Kridin et al. (106) compared over 50,000 patients with AA to a matched-control group and found that the comorbidity between AA and AD was more pronounced than that with other autoimmune diseases. This association was particularly strong in individuals under 20 years of age. Further supporting evidence comes from a case series by Huang et al. (107) in which approximately 70% of patients with AA treated with dupilumab experienced more than 50% hair regrowth, with some also developing white hair regrowth. This accumulating clinical evidence has been further validated in randomized trials. In a phase 2a clinical study conducted by Guttman-Yassky et al. (108), 48 weeks of dupilumab treatment resulted in 22.5% of patients achieving SALT50 (≥50% scalp hair regrowth) and 15% achieving SALT75 (≥75% regrowth). Notably, among patients with baseline serum IgE levels ≥200 IU/ml, these response rates increased significantly to 46.2% and 38.5%, respectively. These findings suggest that Th2 activation may play a pathogenic role in a subset of patients with AA and that serum IgE could potentially serve as a predictive biomarker for treatment responsiveness.

In summary, biologics such as dupilumab, which target the Th2 pathway, may offer meaningful therapeutic alternatives for patients with AA, particularly those unresponsive to conventional treatments or with coexisting atopic conditions.

### 4.6 Mesenchymal stem cells

Mesenchymal stem cells (MSCs) can be derived from various tissues, including skin fibroblasts, peripheral blood, bone marrow, adipose tissue, and tonsils. Regardless of their tissue of origin, MSCs have immunomodulatory properties. Owing to their immunosuppressive nature, allogeneic administration of MSCs does not trigger immune rejection. This unique feature has led to active exploration of both autologous and allogeneic MSCs as therapeutic agents for various refractory autoimmune diseases (109). Notably, encouraging clinical outcomes have been reported in the treatment of several autoimmune diseases (110). AA, classified as an autoimmune disorder, has recently gained attention as a potential target for MSC-based immunomodulatory therapies. A growing body of *in vitro* and *ex vivo* studies suggests that MSCs may serve as a promising treatment option for AA. For instance, Byun et al. (111) demonstrated that pretreatment with MSCs in a mouse model successfully prevented AA induction. Similarly, Li et al. (112) reported that a single exposure of peripheral blood from patients with AA to MSCs via extracorporeal circulation led to sustained hair regrowth that persisted for 1–2 years. Additionally, a study by Park et al. (109) using an animal model showed that localized MSC therapy significantly reduced the expression of proinflammatory cytokines in the skin, including JAK1, JAK2, STAT1, STAT3, IFN-γ receptor, IL-1β, IL-16, IL-17α, and IL-18. Furthermore, the treatment normalized the expression of Wnt/β-catenin signaling pathway genes (LEF1 and β-catenin) and growth factors (FGF7 and FGF2), which are essential for regulating the hair follicle cycle. Collectively, these findings suggest that MSCs have therapeutic potential for the treatment of AA. However, further comprehensive studies are necessary to fully elucidate their mechanisms of action, long-term efficacy, and safety profile.

### 4.7 Potential novel therapeutic targets and therapies

Sirtuin 1 (SIRT1) is a class III histone deacetylase that depends on NAD^+^ and plays a pivotal role in regulating various cellular functions across multiple cell types and tissues. Recent studies have identified SIRT1 as a critical modulator of both innate and adaptive immune responses (113). Previous studies have demonstrated that SIRT1 expression is significantly downregulated in the scalp of patients with AA, particularly within the outer root sheath (ORS) of affected hair follicles. Reduced mRNA and protein levels of SIRT1 have been associated with longer disease duration and histological features consistent with chronic AA subtypes (114). SIRT1 inhibition upregulates the expression of NKG2D ligands and enhances the production of proinflammatory cytokines in ORS cells. Additionally, SIRT1 suppression activates the NF-κB and STAT3 signaling pathways in both ORS cells and C3H/HeJ mice (114). Begum et al. (115) demonstrated that using antisense oligonucleotides to target microRNAs that suppress SIRT1 leads to increased SIRT1 expression and improvement of AA symptoms. These findings suggest that SIRT1 is a promising therapeutic target for the treatment of AA.

Receptor-interacting serine/threonine kinase 1 (RIPK1) is a critical mediator of cell death signaling pathways and plays a central role in regulating inflammatory responses. It is primarily activated downstream of tumor necrosis factor receptor 1 (TNFR1) (116). Aberrant RIPK1 regulation has been implicated in various inflammatory and autoimmune diseases (117). Notably, RIPK1 is also expressed in hair follicles and contributes to hair cycle regulation. RIPK1 inhibition has been shown to promote the transition from the telogen to anagen phase and prolong the duration of the anagen phase (116). Kim et al. (118) recently reported that RIPK1 expression, both at the mRNA and protein levels, was significantly upregulated in the skin of AA mouse models. Single-cell RNA sequencing and immunohistochemistry revealed elevated RIPK1 expression, particularly in DCs and CD8^+^ T cells. Pharmacological inhibition of RIPK1 using agents, such as Necrostatin-1s and GSK2982772, delayed disease onset in an AA mouse model and led to reduced DC and CD8^+^ T-cell infiltration in the skin. Furthermore, in a hair organ culture system mimicking AA, treatment with RIPK1 inhibitors resulted in increased hair shaft elongation. Collectively, these findings suggest that RIPK1 contributes to AA pathogenesis by modulating immune cell activity and that RIPK1 inhibitors may have therapeutic potential for preventing disease onset.

OX40 is a key co-stimulatory molecule essential for the proliferation and survival of T cells. Because it is broadly expressed across various T-cell subsets, it has emerged as an appealing therapeutic target for T-cell–mediated diseases (119). Clinical trials targeting OX40 for the treatment of certain inflammatory skin conditions are currently progressing. According to a study by Fujita et al. (120), OX40 signaling plays a crucial role in cytotoxic T cells by enhancing CD25 expression and IFN-γ production, particularly when naïve CD8^+^ T cells are co-cultured with PBMCs *in vitro*. MCs expressing OX40L are thought to contribute to AA pathogenesis. Increased numbers of OX40L^+^ MCs have been observed in the perifollicular region of samples from patients with AA. Immunohistochemical analysis also revealed close spatial association between CD8^+^ T cells and OX40L^+^ MCs (52). More recently, studies have shown that upregulation of OX40 and OX40L in the skin of patients with AA is accompanied by elevated levels of circulating OX40^+^ and OX40L^+^ leukocytes, regardless of a history of AD (121). These findings suggest that the OX40/OX40L axis is a promising and novel therapeutic target for AA.

The Ikaros family zinc finger 1 (*IKZF1*) gene encodes Ikaros, a zinc-finger transcription factor that plays an essential role in the development and regulation of hematopoietic and immune cells. *IKZF1* is involved in the differentiation and function of various immune cells, including T cells, B cells, NK cells, and neutrophils, and is a key factor in maintaining immune homeostasis (122). Arakawa et al. (123) observed that *IKZF1* overexpression in a mouse model led to increased expression of NKG2D ligands, activation of CD8^+^ T cells, and onset of hair loss in lesional areas. Furthermore, increased expression of the Ikaros protein has also been reported in the lesional skin of patients with AA. These findings suggest that *IKZF1*/Ikaros contributes to the pathogenesis of AA by promoting immune cell activation, perifollicular infiltration, and activation of the NKG2D pathway. Therefore, *IKZF1* could be considered a potential therapeutic target in future AA treatment strategies; however, further research is needed to validate this possibility.

Peroxisome proliferator–activated receptor α (PPARα) is a ligand-activated transcription factor belonging to the NR1C nuclear receptor subfamily, known to play a key role in lipid metabolism across various tissues, as well as in immune regulation, keratinocyte differentiation, lipid synthesis, and skin inflammation (124). Studies have reported that mice deficient in PPARα exhibit prolonged inflammatory responses (124). PPARα is expressed in both the dermal and epithelial cells of human hair follicles (125), and its expression is decreased in the lesional skin of both human and mouse models of AA (126). According to a study by Xuan et al. (126), treatment with a PPARα agonist in a mouse model of AA led to a reduction in CD45^+^, NKG2D^+^, and IFN-γ^+^ CD8^+^ T cells, along with suppression of inflammatory cell infiltration and decreased expression of MHC class I and II molecules. These findings suggest the potential of PPARα agonists as a novel therapeutic approach for AA.

As described above, emerging molecular targets such as SIRT1, RIPK1, OX40, IKZF1, and PPARα appear to contribute to the onset and progression of AA by regulating the hair cycle, immune cell activation, and inflammatory signaling pathways. Therapeutic strategies targeting these molecules may offer promising new treatment options in the future. Further clinical studies on these targets are expected to contribute to the development of novel therapeutic agents (**Table 2**).

**Table 2:** Summary of clinical advantages and therapeutic limitations of emerging AA treatments.

Therapy/Target	Advantages	Limitations
JAK inhibitors	Strongest current clinical evidence; FDA-approved; long-term efficacy	High relapse rate after discontinuation
Low-dose IL-2	Therapeutic potential demonstrated in multiple autoimmune diseases	Short half-life; insufficient efficacy as a monotherapy; precise dosing required; risk of off-target activation
Cytokine/receptor blockade (e.g., anti-NKG2D, anti-IL-12/15/18, CXCL12 blockade)	High selectivity for AA related target	Human efficacy not proven; long-term safety unclear
S1P receptor modulators	Directly blocks immune cell trafficking; Low likelihood of prolonged immunosuppression after drug discontinuation	Need for additional clinical validation
IL-4Rα blockade (Dupilumab)	FDA-approved for other dermatologic conditions; Useful in AA with comorbid AD	Need for additional clinical validation
Mesenchymal Stem Cells	Long-lasting immunosuppression; No immune rejection	Limited clinical validation; mechanisms unclear
Potential novel therapeutic targets (SIRT1, OX40/OX40L, RIPK1, IKZF1, PPARα, etc.)	Provide novel therapeutic targets for patients unresponsive to conventional therapies	Efficacy largely unproven in humans

## 5 Conclusion and future perspectives

**Figure 2:**
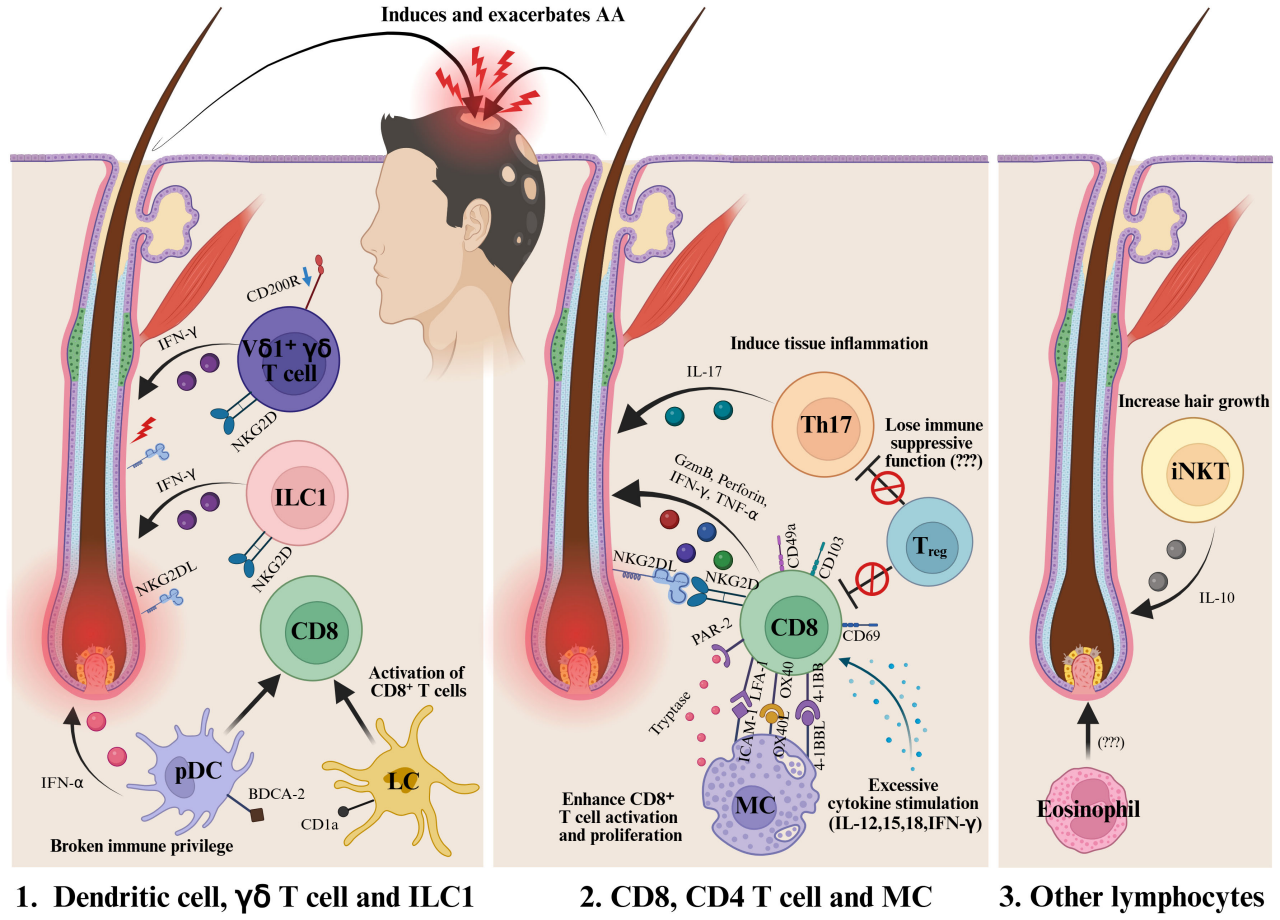
Various immune cells are involved in the onset and progression of AA. In the early stages of the disease, DCs, ILC1s, and γδ T cells are presumed to contribute to the collapse of immune privilege and promote the activation of CD8^+^ immune cells. Among them, CD8^+^ NKG2D^+^ T cells are recognized as the main effector cells in AA. In addition, Th17 cells and MCs have also been reported to play a role in the development and progression of the disease. The loss of the immunosuppressive function of T_regs_ is another area of active investigation. Furthermore, iNKT cells are known to promote hair growth through the expression of IL-10. Further research is required to clarify the role of eosinophils in AA. DC, Dendritic cells; MC, Mast cells; ILC1, Type 1 innate lymphoid cells; Treg, Regulatory T cell; iNKT, Invariant natural killer T cell. Created in BioRender (Agreement number: TX28WE80YA).

AA is an autoimmune disorder primarily driven by the collapse of immune privilege in hair follicles and the subsequent activation of cytotoxic immune responses, particularly involving NKG2D^+^ CD8^+^ T cells. Recently, the roles of innate immune cells such as MCs, DCs, ILC1, and γδ T cells in AA pathogenesis have received increasing attention (**Figure 2**). These findings highlight the potential for developing cell-specific or combination immunotherapies targeting these innate immune populations.

Significant progress in both preclinical animal models and human clinical studies has greatly advanced our understanding of the immunopathogenesis of AA, paving the way for the development of various immunomodulatory therapies (**Table 3**). Among the developed treatments, JAK inhibitors have demonstrated remarkable efficacy in patients with moderate-to-severe AA, and JAK1/2 and JAK3 inhibitors are now widely used as systemic therapies. However, the high relapse rate after treatment discontinuation and concerns about long-term safety due to systemic immunosuppression highlight the continuing need for safer and more durable treatment strategies. Relapse following the discontinuation of JAK inhibitors remains a major clinical challenge in the management of AA, and no definitive solution has yet been established. Although the JAK–STAT signaling pathway plays a pivotal role in the pathogenesis of AA, additional immunological mechanisms are likely to contribute to disease onset and recurrence. In particular, the progression and recurrence of several skin inflammatory diseases such as vitiligo, psoriasis, and AD have been closely associated with T_RMs_ (127, 128), and studies using AA mouse models have demonstrated the presence of T_RMs_ expressing CD69, CD49a, and CD103 within the skin (22, 23).

**Table 3:** Therapeutic agents newly investigated for the treatment of AA.

Drug	Classification	Targets of action	Phase	Clinical trials ID
Baricitinib	JAK inhibitors approved by the FDA for use in AA	JAK 1/2	2/3 3	NCT03570749 NCT03899259
Deuruxolitinib		JAK 1/2	3 3	NCT04518995 NCT04797650
Ritlecitinib		JAK 3/TEC	2a 2b/3 3	NCT02974868 NCT03732807 NCT04006457
Upadacitinib	JAK inhibitor	JAK 1	3	NCT06012240
Ivarmacitinib (SHR0302)		JAK 1	2 3	NCT04346316 NCT05470413
KL130008		JAK 1/2	2	NCT05496426
Ifidancitinib		JAK 1/3	2	NCT03759340
Tofacitinib		JAK1/2/3	4	NCT03800979
Jaktinib		JAK 1/2/3	2 3	NCT04034134 NCT05051761
SYHX1901		JAK/SYK	2	NCT06562894
Brepocitinib (PF-06700841)		JAK1/TYK2	2	NCT05076006
Deucravacitinib		TYK2	2	NCT05556265
Small dose IL-2	Cytokine therapy	T_reg_ expansion	1/2	NCT01840046
EQ101 (BNZ-1)	Antibody therapy	IL-2, IL-9 and IL-15	2	NCT05589610
Anti-12/15/18		IL-12, IL-15 and IL-18	–	Not applicable
Anti-NKG2D		NKG2D	–	Not applicable
Anti-CXCL12		CXCL12	–	Not applicable
Dupilumab (Anti-IL-4Rα)		IL-4 and IL-13	2	NCT03359356
Etrasimod	S1PR modulator	S1PR 1,4 and 5	2	NCT04556734
NXC736		S1PR 1 and 4	2	NCT06104839
MSC	Stem cell therapy	Anti-inflammatory effect	–	Not applicable
Antisense oligonucleotides	MicroRNA therapy	microRNAs that suppress SIRT1	–	Not applicable
RIPK1 inhibitors	Inhibitor therapy	RIPK1	–	Not applicable
PPARα agonist	Agonist therapy	PPARα	–	Not applicable

The role of IL-15 in the formation, regulation, and maintenance of T_RMs_ is also of great importance (129–131). In vitiligo, a CD8^+^ T cell–mediated autoimmune skin disease, short-term intradermal administration of an anti-CD122 (IL-15Rβ) antibody has been reported to induce a sustained therapeutic effect even after treatment discontinuation (132). Since such intradermal approaches are already widely utilized in dermatological practice, targeting the T_RM_–IL-15 axis through monotherapy or combination therapy with JAK inhibitors may represent a promising and durable therapeutic strategy that could prevent disease relapse and achieve long-term disease control while maintaining a favorable safety profile in AA. Furthermore, novel molecular targets such as SIRT1, RIPK1, OX40, *IKZF1*, and PPARα have been identified as regulators of immune cell activation, inflammatory signaling, and hair cycle modulation, suggesting their potential as therapeutic targets for the development of next-generation treatments for AA. Altogether, these emerging therapeutic approaches should be evaluated not only for their ability to induce clinical improvement but also for their potential to prevent relapse and achieve sustained disease remission in future studies.

Given the heterogeneity of treatment responses, particularly among patients who are refractory to conventional therapies, the development of personalized treatment algorithms is becoming increasingly important. For instance, significant improvement in scalp IFN-related biomarkers has been reported following treatment with ritlecitinib and brepocitinib (133), suggesting that JAK inhibitors may be more effective in patients with a dominant IFN-γ/CD8^+^ T cell axis and elevated expression of IFN-inducible chemokines such as CXCL9 and CXCL10. In contrast, patients with AA accompanied by AD and baseline serum IgE levels exceeding 200 IU/mL have been reported to show favorable clinical responses to dupilumab (108). These findings indicate that in AA patients presenting with allergic comorbidities such as AD, allergic rhinitis, or asthma, or in those exhibiting a Th2-skewed immune response characterized by elevated Th2-related chemokines and cytokines (e.g., TARC/CCL17), IL-4Rα–blocking agents like dupilumab may represent a more effective therapeutic alternative. Such observations underscore the potential utility of serologic and transcriptional biomarkers in guiding treatment selection and response prediction. Moreover, advances in multi-omics and transcriptomic profiling technologies are expected to facilitate patient stratification based on cytokine signatures and immune cell composition, further promoting the realization of personalized therapy for AA in the near future.

In conclusion, although considerable progress has been made in elucidating the mechanisms underlying AA, further research is needed to translate this knowledge into effective and long-lasting therapies. Continued efforts in this direction have the potential to establish safe, sustainable, and individualized treatment strategies, ultimately improving the quality of life of individuals affected by this complex disease.
